# Unveiling the nature of atomic defects in graphene on a metal surface

**DOI:** 10.3762/bjnano.15.37

**Published:** 2024-04-15

**Authors:** Karl Rothe, Nicolas Néel, Jörg Kröger

**Affiliations:** 1 Institut für Physik, Technische Universität Ilmenau, D-98693 Ilmenau, Germany

**Keywords:** atomic force microscopy and spectroscopy, graphene, scanning tunneling microscopy and spectroscopy

## Abstract

Low-energy argon ion bombardment of graphene on Ir(111) induces atomic-scale defects at the surface. Using a scanning tunneling microscope, the two smallest defects appear as a depression without discernible interior structure suggesting the presence of vacancy sites in the graphene lattice. With an atomic force microscope, however, only one kind can be identified as a vacancy defect with four missing carbon atoms, while the other kind reveals an intact graphene sheet. Spatially resolved spectroscopy of the differential conductance and the measurement of total-force variations as a function of the lateral and vertical probe–defect distance corroborate the different character of the defects. The tendency of the vacancy defect to form a chemical bond with the microscope probe is reflected by the strongest attraction at the vacancy center as well as by hysteresis effects in force traces recorded for tip approach to and retraction from the Pauli repulsion range of vertical distances.

## Introduction

Defects in lattices of two-dimensional (2D) materials are considered as promising building blocks for tailoring electronic and phononic band structures, magnetic texture, photon emission, and charge carrier concentration [1]. In addition, defects profoundly impact, in a beneficial or detrimental manner, characteristic properties of 2D materials [2].

A prominent 2D material is graphene. Intact graphene, the 2D sp^2^ arrangement of C atoms in a honeycomb mesh, is well known for its appealing electronic and mechanical properties [3–4]. However, during its epitaxial growth in surface science experiments or its fabrication for applications, defects, that is, deviations from the ideal 2D lattice, inevitably occur. Examples for defects are vacancies, interstitial atoms, grain boundaries, stacking faults or wrinkles [5–23]. Even single missing C atoms were demonstrated to severely change electronic [11,13–14,18], mechanical [17], and magnetic [7–8,10,12] characteristics. It is therefore not surprising that the intentional creation of defects, which has mainly been achieved by noble-gas ion irradiation [6,13–14,17,19,21,24], represents an opportunity for systematic defect studies.

The work presented here was stimulated by the lack of experimental data on the actual geometry of atomic-scale defects in graphene. So far, scanning tunneling microscope (STM) topographies have been claimed to be in accordance with, for example, single-C vacancy sites. However, clear-cut evidence for a missing C atom in the graphene lattice has remained elusive. Therefore, in addition to an STM, an atomic force microscope (AFM) has been used in the present study to unveil the geometric structure of the defect sites. Surprisingly, the smallest defect in graphene on Ir(111), which appears as a depression in STM images and, therefore, may readily be assigned to a single-C vacancy site, gives rise to an undistorted graphene lattice in AFM images. In contrast, slightly larger defects are indeed lacking the graphene atomic lattice structure in their interior. Spatially resolved spectroscopy of the differential conductance (d*I*/d*V*, *I*: tunneling current, *V*: bias voltage) and of the tuning fork resonance frequency change (Δ*f*) further unravel marked differences between these two kinds of defects.

## Experimental

A combined STM-AFM was operated in ultrahigh vacuum (5 × 10^−9^ Pa) and at low temperature (5 K). Surfaces of Ir(111) were cleaned by Ar^+^ ion bombardement and annealing. The epitaxial growth of graphene proceeded by exposing the heated (1300 K) Ir(111) surface to the gaseous molecular precursor C_2_H_4_ (purity: 99.9%) at a partial pressure of 10^−5^ Pa for 120 s [25–26]. Atomic-scale defects were created by bombarding graphene-covered Ir(111) with low-energy (140 eV) Ar^+^ ions (purity of the Ar gas: 99.999%) [27–30] at room temperature for 5 s followed by annealing (900 K, 5 min). The Ar^+^ beam enclosed an angle of 15° with the surface normal and exhibited a flux of ≈0.01 1/(nm^2^·s). A chemically etched (NaOH, 0.1 M) W wire (purity: 99.99%, diameter: 50 μm) was used as the tip material. Tips were cleaned by field emission on and indentations into a clean Au(111) crystal and, presumably, coated with a Au film. The tip shape was further sharpened by single-atom transfers from the tip to the sample surface [31–36]. Topographic STM data were recorded in constant-current as well as constant-height modes with the bias voltage applied to the sample. Constant-height scanning tunneling spectroscopy (STS) of d*I*/d*V* was performed by sinusoidally modulating (5 mV_rms_, 725 Hz) the dc bias voltage and measuring the first harmonic of the ac current response of the tunneling junction with a lock-in amplifier. For AFM data acquisition, resonance frequency changes of an oscillating piezoelectric tuning fork sensor [37–38] (resonance frequency: 30.5 kHz, quality factor: 45000, amplitude: 50 pm) were mapped at constant height for topographic images. The vertical force between tip and sample was extracted from distance-dependent measurements of the resonance frequency shift [39–40]. Topographic STM and AFM data were processed using WSxM [41].

## Results and Discussion

### Scanning tunneling microscopy and spectroscopy findings

**Figure 1:**
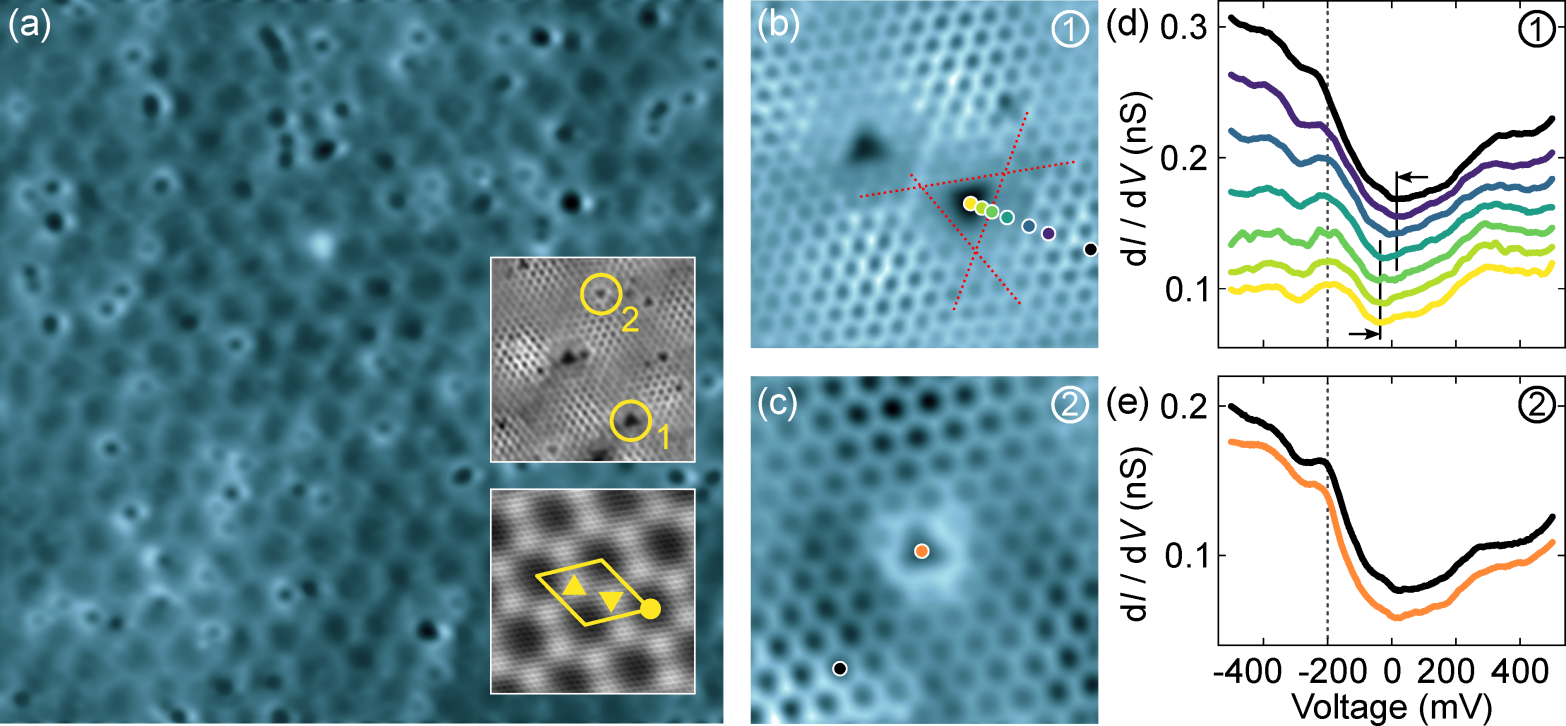
Scanning tunneling microscopy and spectroscopy of defect types **1** and **2** in graphene on Ir(111). (a) Constant-current STM image of Ar^+^-bombarded graphene (bias voltage: 100 mV, tunneling current: 50 pA, size: 40 nm × 40 nm). Top inset: atomically resolved graphene lattice with defects **1** and **2** (10 mV, 40 nA, 5.9 nm × 5.9 nm). Bottom inset: moiré superstructure with lozenge unit cell (side length: ≈2.53 nm) and the atomically resolved graphene lattice. Triangles mark different valley stacking domains with ▲ and ▼ tentatively marking hcp and fcc stacking, while the dot (●) indicates the mound top site of the moiré lattice (10 mV, 5 nA, 8 nm × 8 nm). (b) Two adjacent type-**1** vacancies located at an hcp (left) and an fcc (right) position of the moiré lattice (10 mV, 55 nA, 3.6 nm × 3.6 nm). Red dashed lines approximate the defect edges (see text). (c) Single type-**2** defect at an fcc valley site (10 mV, 25 nA, 2.5 nm × 2.5 nm). The gray scale (from dark to bright) covers apparent heights from 0 to (a) 190 pm, (b) 50 pm and (c) 30 pm. (d) Series of constant-height d*I*/d*V* spectra recorded at the positions marked by the colored dots in (b). The steplike feature at ≈−200 mV (dashed line) corresponds to an Ir(111) surface resonance. The solid vertical lines mark the shift of the suggested Dirac cone signature. (e) Spectra of d*I*/d*V* recorded atop the positions indicated by the orange and black dots in (c). The STS data for defects **1** and **2** were acquired with the same tip. The spectra are shifted vertically by 0.02 nS. Feedback loop parameters prior to spectroscopy: 500 mV, 50 pA.

After gentle Ar^+^ ion bombardment, graphene-covered Ir(111) gives rise to STM images as depicted in Figure 1a. The periodic superstructure of depressions with a measured repeat distance of 2.59 ± 0.05 nm reflects the moiré pattern that is caused by the lattice mismatch between graphene and Ir(111), where graphene 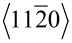 directions are aligned with 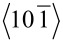 directions of the metal substrate [25,42–45]. The lozenge unit cell of the coincidence lattice is depicted in the bottom inset to Figure 1a, which shows an atomically resolved STM image of graphene. Mounds of the moiré pattern appear as depressions, while valleys show bright contrast at the specific tunneling parameters used for the STM topograph in Figure 1a. At higher tunneling currents, a contrast inversion occurs (top inset to Figure 1a), and mounds (valleys) appear bright (dark). A similar contrast inversion was previously reported for different tunneling voltages [42] and associated with the specific electronic structure of graphene on Ir(111) [45]. In the present experiments, the contrast inversion is induced by the reduction of the tip–graphene separation. As will be shown below, the involved junction currents correspond to a separation that is still larger than, but close to, the point of maximum attractive force. Therefore, a tentative rationale to the observed contrast inversion is the increased tip–graphene hybridization compared to the far tunneling range, which may entail a modification of the graphene electronic structure or enhance the contribution of substrate states to the junction current [46]. The mounds and adjacent valleys of the moiré superstructure are characterized by different graphene–Ir(111) stackings. Mounds of the moiré superstructure correspond to C hexagons of the graphene lattice residing atop an Ir atom, while adjacent valleys of the moiré superstructure are associated with C hexagons residing atop an hexagonal closed-packed (hcp) and a face-centered cubic (fcc) site of Ir(111). From STM images alone, hcp and fcc valleys cannot be distinguished, that is, the assignment in Figure 1a is tentative. In addition to the moiré superlattice, depressions with various sizes and shapes are visible, which are not present on the freshly prepared graphene surface (Supporting Information File 1, Figure S1). Therefore, the depressions are associated with defects of the graphene lattice induced by the Ar^+^ ion impact. The most abundant defect types (≈58% of all observed defects) exhibit a triangular shape (top inset to Figure 1a). Type **1** (≈35%, Figure 1b) appears with a laterally larger and deeper depression than type **2** (≈23%, Figure 1c). Moreover, type-**1** defects occur nearly exclusively (≈100%) at valley sites of the moiré lattice. The hcp and fcc stacking of the valley sites defines the orientation of the triangular shape of the defect, pointing in opposite directions at the two valley sites [24]. Defects of type **2** do not show a preferential moiré lattice site (top inset to Figure 1a). Other defects observed in the STM images are most likely due to Ir(111) surface impurities. These defects exhibit different shapes and contrasts in STM images and are already present on clean graphene-covered Ir(111) (Supporting Information File 1, Figure S1).

Figure 1b presents a close-up STM view of the first kind of equilateral triangular defects. The edges are oriented along the symmetry directions of the graphene lattice. To estimate the edge lengths, an equilateral triangle was circumscribed (dashed lines) that continues the edges of those C hexagons of intact graphene that are closest to the defect. A length of 0.65 ± 0.05 nm has been inferred in this manner. The apparent depth of the defect at 10 mV is 28 ± 9 pm. The second type of triangular defects (Figure 1c) exhibits a smaller side length of 0.46 ± 0.03 nm with the same orientations as observed for **1**. The apparent depth of type-**2** defects at 10 mV is 7 ± 3 pm. The uncertainty margins reflect the standard deviation of measured lengths of ten different defects of each type. Importantly, from STM data alone, both triangular defects appear as depressions with no identifiable interior structure. Therefore, they may be interpreted as graphene vacancy sites, that is, as sites with missing C atoms.

As shown by the spectra of d*I*/d*V* for the two defect types (Figure 1d,e) the electronic structure differs. Atop the center of **1** (bottom spectrum of Figure 1d) two prominent signatures contribute to the spectral data. The broad steplike variation at approx. −200 mV (dashed line) is due to the Ir(111) surface resonance at the 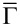-point of the surface Brillouin zone (BZ) [47], which is shifted toward the Fermi energy (*E*_F_) because of the presence of graphene [48]. The 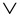-shaped feature with minimum signal slightly below zero bias may be associated with the Dirac cone at the BZ 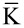-point. Figure 1d reveals the spatial evolution of the spectra, which shows a gradual quenching of the Ir(111) surface resonance signal accompanied by a small shift toward zero bias voltage upon laterally approaching the defect center. The Dirac cone signal shifts from ≈18 mV above undistorted graphene (top spectrum in Figure 1d) to ≈−40 mV atop the center of the defect. While the extracted energy of the Dirac point is in agreement with previously reported values from STS experiments [49–51], it is lower than the energy observed in photoemission experiments [52]. A possible rationale is the locally lifted graphene in the presence of the tip [53], which in turn decreases the charge transfer from graphene to the metal and reduces the p-doping [52] and concomitantly causes a lowering of the Dirac point energy. Type-**1** defects exhibit the same behavior in spatially resolved STS measurements, independent of the moiré valley they reside at. In contrast, d*I*/d*V* data acquired above **2** are essentially identical to spectroscopic data of pristine graphene (Figure 1e). The lateral evolution of the d*I*/d*V* spectra hints at a markedly different character of defects **1** and **2**, which will further be explored on the basis of the AFM results to be discussed below. They will clarify structural aspects of the defects and help understand the different spectroscopic properties.

Before presenting the AFM results, a comparison of the defect spectra in Figure 1d,e with previous results obtained for atomic-scale defects in graphene on other surfaces is noteworthy. Very pronounced electronic resonances localized at vacancy defects were reported for graphite surfaces [13], graphene on Pt(111) [14], and SiC(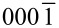) [16]. These resonances were interpreted as a collective excitation of the graphene π orbitals near the void [54], which depends on the coupling of the C atoms to the substrate surface. Therefore, the graphene–surface hybridization plays an important role in the occurrence of this resonance. Indeed, the resonance was not observed at all graphene defects on Pt(111). At some sites, it was quenched because of an increased interaction between the defect and the metal [55]. Therefore, the absence of a similar resonance in d*I*/d*V* spectra of graphene defects on Ir(111) may be due to an increased graphene–metal interaction compared with Pt(111), although both graphene–metal hybrid structures belong to the weak-hybridization regime [43]. Another rationale is the deviation of the observed defects **1** and **2** from a monatomic vacancy site, which will further be explored in the following.

### Atomic force microscopy and spectroscopy findings

**Figure 2:**
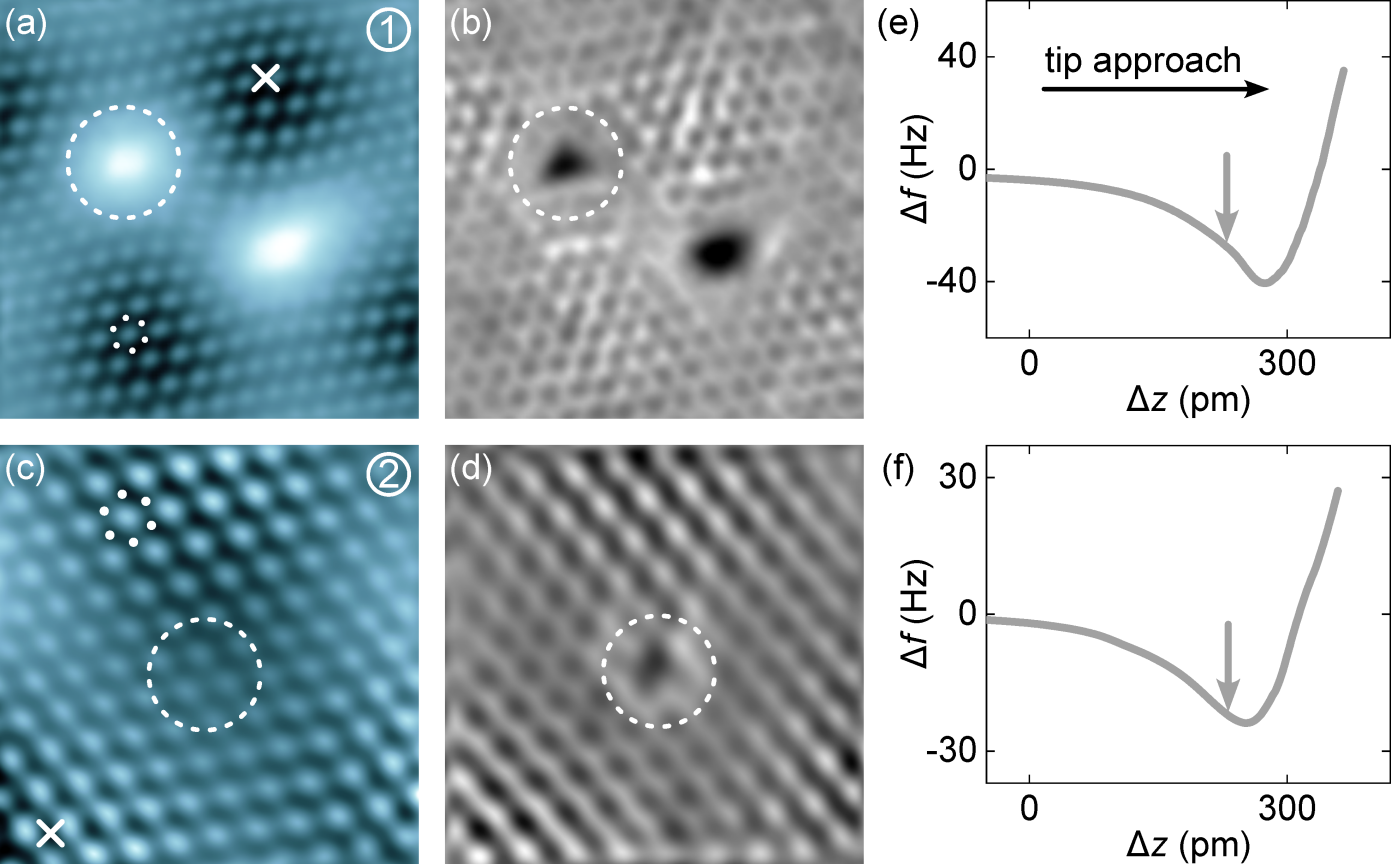
Atomic force and scanning tunneling microscopy of defect types **1** and **2** in graphene on Ir(111). (a) Constant-height AFM image and (b) simultaneously recorded tunneling current map of two adjacent defects (10 mV, 3.6 nm × 3.6 nm). (c),(d) As (a),(b) for a type-**2** defect (10 mV, 2.5 nm × 2.5 nm). The relevant defects in (a–d) are encircled by a dashed line. The gray scale (from dark to bright) covers changes in the resonance frequency shift from (a) −48 to −13 Hz and (c) −36 to −18 Hz as well as changes in the tunneling current from (b) 4 to 19 nA and (d) 4 to 7 nA. (e, f) Variation of Δ*f* with tip displacement Δ*z* (tip approach from left to right) on intact graphene (cross in (a) and (c)). The vertical arrow marks the tip excursion used for the constant-height Δ*f* current maps in (a–d). Displacement Δ*z* = 0 defines the tip–sample distance at which the feedback loop was deactivated above pristine graphene (10 mV, 50 pA).

Figure 2 compares constant-height AFM topographs of the defects (Figure 2a,c) with simultaneously recorded current maps of the same defects (Figure 2b,d). The tip–surface distance for the AFM and current maps was defined by the tip excursions marked with an arrow in the Δ*f* traces on clean graphene (Figure 2e,f). Tip approach, that is, the decrease of the tip–surface distance, is reflected by increasing tip excursions Δ*z* (horizontal arrow in Figure 2e). In Figure 2a, two adjacent defects appear as bright protrusions, where only the encircled defect is of kind **1**. It resides at a valley of the moiré lattice (data for a type-**1** defect at the other valley site are presented in Supporting Information File 1, Figure S2). Its brighter contrast compared to the intact graphene environment in the AFM topograph indicates that the attraction of type-**1** defects is considerably lower than that of intact graphene at the chosen tip–surface distance. In the associated current map (Figure 2b), defect **1** appears as a uniform depression without interior structure. Moreover, the graphene lattice, which appears via the protruding honeycomb cells in the AFM data, is distorted in the vicinity of the defects. The rows of honeycomb cells are not straight and rather follow curved trajectories that are bent towards the defect sites.

The Δ*f* map for a type-**2** defect (Figure 2c) was likewise acquired in the attractive regime (arrow in Figure 2f) where the graphene C atoms (white dots in Figure 2c) appear with lower contrast than the interior of the honeycomb cell [56]. Surprisingly, the AFM topograph of the assumed vacancy site hints at an intact graphene lattice (encircled area in Figure 2c) with no evidence of distortions, which clearly contrasts the result of the current map where defect **2** appears as a depression (Figure 2d).

**Figure 3:**
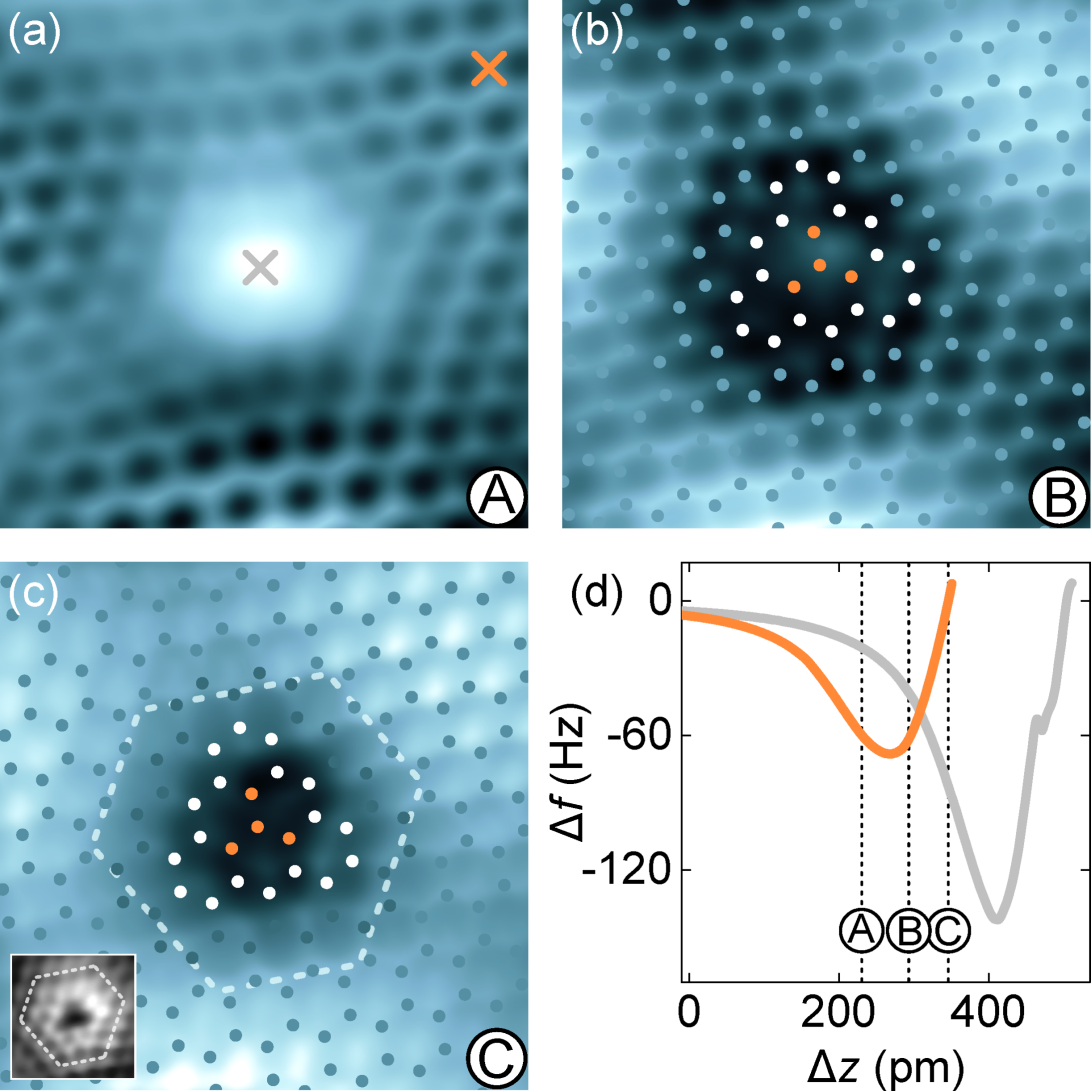
Vertical probe–surface distance dependence of AFM topographies of defect **1**. (a–c) Constant-height AFM images of a type-**1** defect (10 mV, 2.4nm × 2.4 nm). The tip–surface distance decreases from (a) to (c). The gray scale (from dark to bright) encodes resonance frequency changes from (a) −75 to −35 Hz, (b) −85 to −25 Hz, and (c) −120 to 40 Hz. Superimposed dots in (b) and (c) represent positions of C atoms (blue: C atom of intact graphene lattice; white: C atom at the defect edge; orange: missing C atom). White dashed line in (c) represents the edge of the dim rim surrounding the vacancy (see text). Inset to (c): constant-height current map (1.4 nm × 1.4 nm) of a type-**1** defect recorded at a comparable tip excursion as the Δ*f* map in (c). (d) Variation Δ*f*(Δ*z*) acquired atop intact graphene (orange line, top right cross in (a)) and above the defect (gray line, central cross in (a)). Labels **A**–**C** indicate the corresponding tip displacements for the topographic images in (a–c). The displacement Δ*z* = 0 is defined by the feedback loop parameters 10 mV and 50 pA above intact graphene. The same tip–surface distance prior to data acquisition above the defect is ensured by taking the apparent height difference at the feedback loop parameters into account.

Type-**1** defects were further analyzed by Δ*f* maps at different tip–surface distances (Figure 3a–c). The underlying tip excursions **A**–**C** are marked in the Δ*f*(Δ*z*) data sets obtained atop the defect and intact graphene (Figure 3d). The latter data were acquired at a lateral distance of a few graphene lattice constants apart from the defect (top right cross in Figure 3a). Atop the defect center, Δ*f*(Δ*z*) (gray line in Figure 3d) shows a minimum that is considerably lower and that occurs at larger tip excursions than observed for intact graphene.

At tip excursion **A**, defect **1** appears as a protrusion in Δ*f* maps (Figure 3a), while the surrounding graphene lattice exhibits lower Δ*f* contrast, which is in accordance with the Δ*f* behavior displayed in Figure 3d. In particular, the interior of the honeycomb unit cell is dark at this tip–graphene distance. Reaching the tip displacement **B**, which slightly exceeds the Δ*f* minimum, the formerly protruding defect turns into a depression with a weak central protrusion. Owing to the clearly resolved honeycomb mesh of graphene, the C atom positions can be superimposed as dots. The central protrusion of the Δ*f* map coincides with the position of a missing C atom. At even smaller tip–surface distances, which are well within the Pauli repulsion range (**C** in Figure 3d), the graphene honeycomb unit cell exhibits bright contrast, while defect **1** gives rise to a uniform depression without the central protrusion observed for tip excursion **B**. In addition, the depression of the Δ*f* map occupies an extended area that includes the three C atoms that are suggested to embrace the central protrusion in Figure 3b. Therefore, Δ*f* topographs acquired in the Pauli repulsion distance range are indicative of in total four missing C atoms, that is, defect **1** is compatible with a tetravacancy that was previously put forward by calculations accompanying an STM experiment [24]. The boundary of the tetravacancy is most likely lowered toward the metal surface allowing hybridization of the unsaturated C dangling bonds with substrate d bands. This scenario would explain the shift of the Dirac cone signature in d*I*/d*V* spectra from positive sample voltages for intact graphene to negative voltages atop the defect (Figure 1d). While intact graphene on Ir(111) is slightly p-doped and exhibits the Dirac cone at energies above *E*_F_ [52], hybridization of the graphene defect with the metal possibly induces electron transfer into graphene giving rise to local n-doping and the Dirac cone below *E*_F_. In addition, the distortion of the graphene lattice that accompanies the increased hybridization with the surface may explain the dim rim of the vacancy in Δ*f* maps. The rim exhibits a hexagonal shape with nonuniform edge lengths (dashed lines in Figure 3c). It is also present as a blurry fringe in STM images (Figure 1b).

While topographic AFM data of type-**1** defects hint at a tetravacancy site, the actual origin of type-**2** defects remains elusive. The STS and AFM results presented here essentially exclude a monatomic vacancy site. Spectra of d*I*/d*V* recorded atop defect-free graphene and above defect **2** are almost identical (Figure 1e), while AFM topographs show an undistorted graphene lattice (Figure 2c). A tentative rationale is then the presence of an Ar^+^-induced Ir(111) surface defect [57].

**Figure 4:**
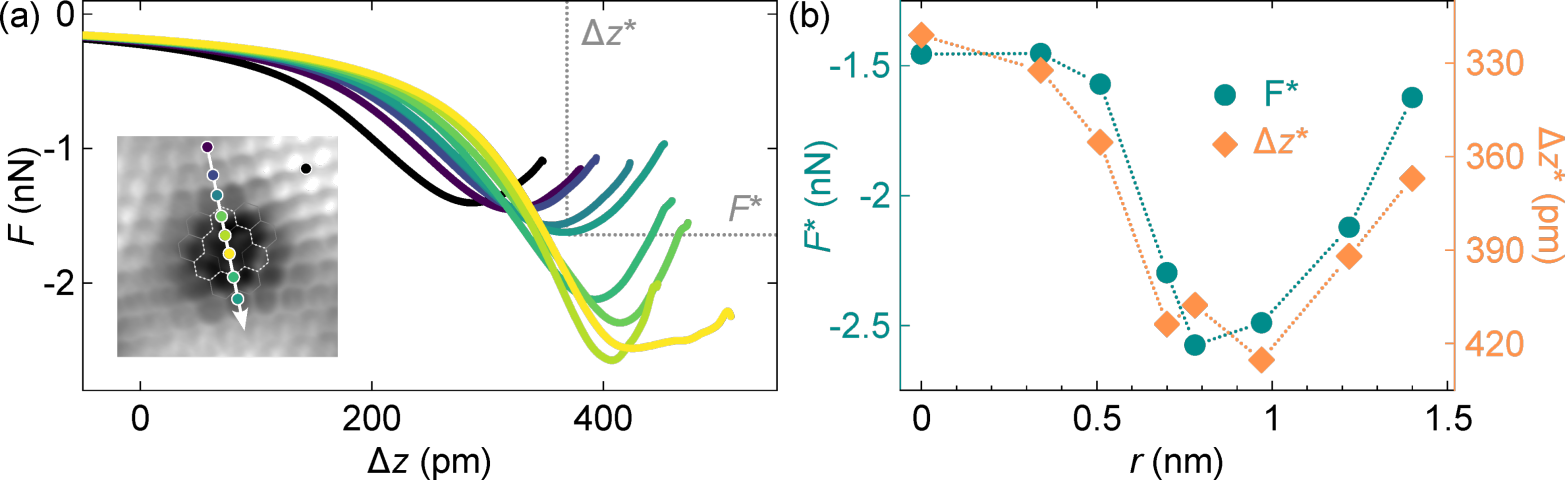
Total vertical force *F* as a function of tip excursion and lateral evolution of the point of maximum attraction (Δz*, F*). (a) Variation of *F* with tip displacement Δ*z* on the indicated path across a type-**1** defect. Displacement Δ*z* = 0 is defined by the feedback loop parameters prior to the measurements (10 mV, 50 pA). Displacement Δz* and the associated force minimum F* are indicated in one data set. Inset: AFM image of a type-**1** defect with marked spectroscopy path oriented along 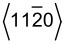 (10 mV, 2.4 nm × 2.4 nm). (b) Evolution of F* (dots) and Δz* (lozenges) as a function of the distance *r* with *r* = 0 at the top side of the path depicted in the inset to (a). Dotted lines serve as guides to the eye.

In a next step, AFM imaging of the defects at different tip–surface distances was complemented by spatially resolved Δ*f*(Δ*z*) measurements (Figure 4). Figure 4a shows the evolution of the total vertical force recorded along the path across a type-**1** defect indicated in the inset. The vertical force *F* is extracted from Δ*f* data following a previously reported algorithm [39–40]. The minima F*, which are defined by the points of maximum attraction attained at Δz* (F* = *F*(Δz*)), shifts towards larger tip excursions Δz* and larger magnitudes |F*| upon laterally approaching the defect center. The evolution of F* and Δz* with the consecutive positions along the path (from top to bottom) is depicted in Figure 4b. The strongest attraction 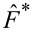 ≈ −2.6 nN and largest tip excursion 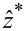 ≈ 425 pm are observed at the defect center (*r* = 0.78 nm). These observations may be rationalized in terms of a more pronounced tendency of the defect center to form a chemical bond with the tip. In the case of defect **1**, dangling C bonds are most likely saturated by their hybridization with the Ir(111) surface. However, the proximity of a metal tip may offer a preferred bonding partner. A similar conclusion was inferred from spatially resolved force spectroscopy across a phthalocyanine molecule on Ag(111) whose pyrrolic H atoms had been removed. Despite the bonds formed by the H-detached N atoms of the molecule with the metal substrate, an increased reactivity was reported [58]. Intact graphene on Ru(0001) was previously demonstrated to exhibit locally different chemical reactivity on the basis of current-versus-distance characteristics [59]. For the second kind of defects, spatially resolved vertical-force traces are nearly identical and, therefore, do not hint at varying differences in bond formation (Supporting Information File 1, Figure S3).

**Figure 5:**
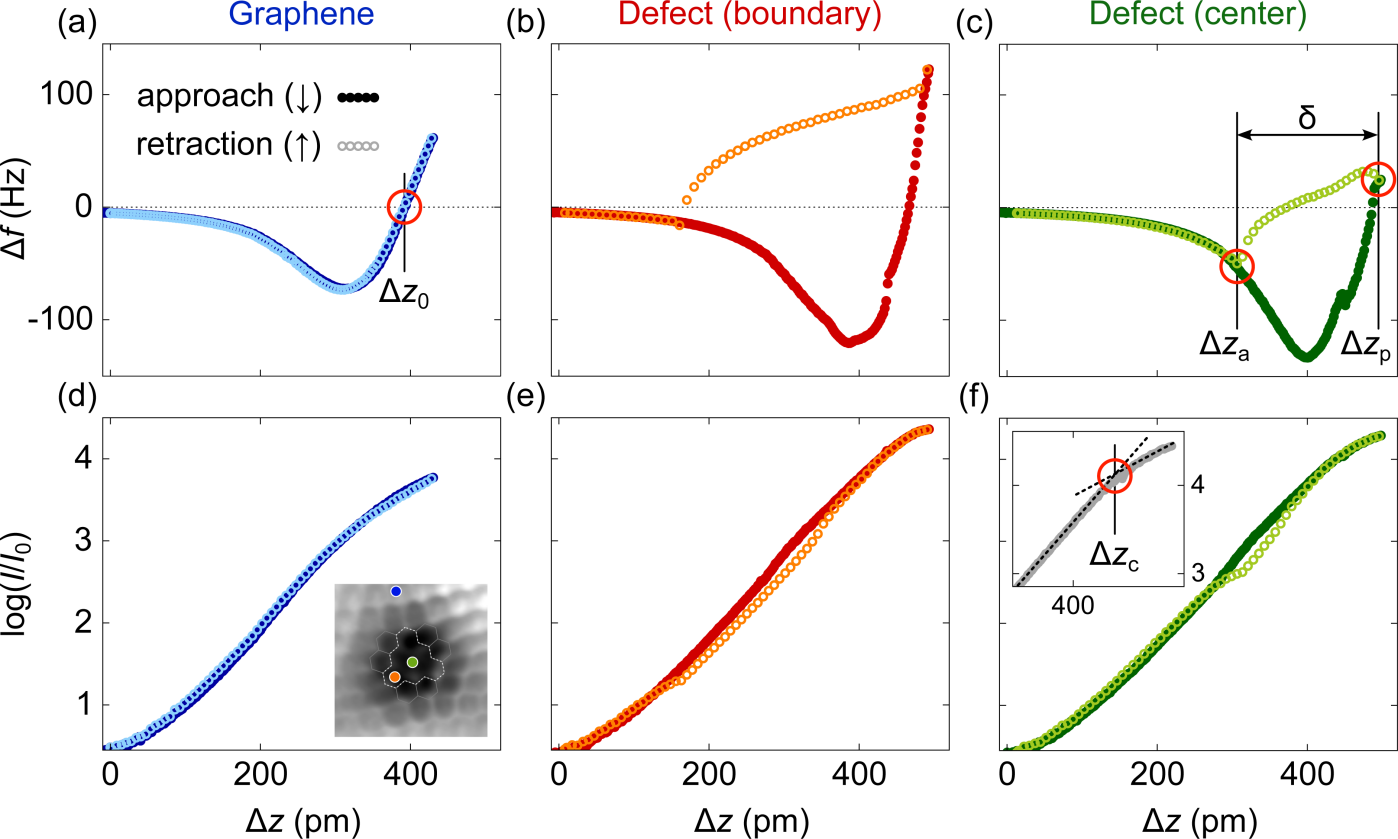
Hysteresis loops in approach (filled symbols) and retraction (open symbols) Δ*f* and *I* traces. Variations Δ*f*(Δ*z*) on (a) pristine graphene as well as atop (b) the defect-**1** boundary and (c) its interior. The zero of Δ*f* in (a) is marked Δ*z*_0_ and corresponds to the point of maximum attraction. The width of the hysteresis loop (δ) is defined by the difference of tip displacements associated with the intersections of Δ*f* approach and retraction traces in (e). (d–f) As (a–c), for *I*(Δ*z*) simultaneously recorded with Δ*f*(Δ*z*). Zero tip displacement is defined by the feedback loop parameters 10 mV and *I*_0_ = 50 pA. Inset to (d): Constant-height Δ*f* map of defect **1** (10 mV, 1.8 nm × 1.8 nm) with indicated positions for Δ*f*(Δ*z*) and *I*(Δ*z*) data acquisition.

The propensity to form a chemical bond between the Au tip apex atom and C atoms close to the center of type-**1** defects is likewise reflected by the occurrence of hysteresis loops in Δ*f* and *I* approach and retraction traces. Figure 5 compares Δ*f* variations for tip approach (dots, Δ*f*↓) and retraction (circles, Δ*f*↑) on pristine graphene (Figure 5a) as well as on the boundary (Figure 5b) and the center (Figure 5c) of a type-**1** defect. The positions of data acquisition are marked in the inset to Figure 5d. While for graphene Δ*f*↓ and Δ*f*↑ data coincide, the Δ*f* traces for the tetravacancy exhibit a hysteresis loop in tip approach and retraction cycles. The width of the loop, defined as δ = Δ*z*_p_ − Δ*z*_a_ with Δ*z*_a_ and Δ*z*_p_ denoting the tip excursions where Δ*f*↓ and Δ*f*↑ intersect (Figure 5c), varies across the defect. Large values of δ are mostly observed at the boundary and in the central part of the proposed tetravacancy. Hysteresis widths exceeding 300 pm with a maximum of ≈330 pm were extracted from Δ*f* cycles in these regions. Lower values of δ including vanishing hysteresis loops were mainly probed in the interior part of the vacancy. A spatial map of δ is presented in Figure S4 of Supporting Information File 1.

For the interpretation of these observations, it is helpful to explore the simultaneously recorded *I*(Δ*z*) traces (Figure 5d–f). The hysteretic behavior that is present for Δ*f* at the defect site is likewise observed in approach (*I*↓) and retraction (*I*↑) current data, albeit with a smaller width of the hysteresis loop. The onset of deviations of *I*↓ from a uniform exponential increase, marked Δ*z*_c_ in the inset to Figure 5f, signals the collapse of the tunneling barrier and the formation of a chemical bond between the tip and the surface [32,36,60–61], that is, between a Au and a C atom. In agreement with a previous report [62], Δ*z*_c_ ≈ Δ*z*_0_ with Δ*z*_0_ being the zero of Δ*f*↓ (Figure 5a), that is, the onset of bond formation corresponds to the point of maximum attraction. Importantly, at Δ*z*_c_ ≈ Δ*z*_0_ the equilibrium Au–C bond length has not yet been reached. To this end, the tip has to be displaced further by Δ*z >* Δ*z*_c_ to reach the energy minimum.

The hysteresis loop in Δ*f* and *I* data can, therefore, be rationalized in terms of a Au–C bond that is formed upon approaching the tip to the defect up to the point of maximum attraction at Δ*z*_c_ ≈ Δ*z*_0_. This bond remains intact during further tip approach up to Δ*z*_p_ and during tip retraction. The retraction of the tip may be accompanied by a partial detachment of graphene from the surface. As soon as the mechanical load surpasses the bond strength, it breaks at Δ*z*_a_. A comparable scenario was put forward previously for graphene on SiC(0001), where intact graphene was lifted from the surface after forming a Au tip–graphene bond and retracting the tip [63]. Hysteresis loops in Δ*f* and *I* were then likewise observed. In the findings presented here, intact graphene does not exhibit hysteretic behavior, although one may expect a similar Au–C bond as proposed for graphene on SiC(0001). Most likely, the graphene–Ir(111) coupling is stronger than the graphene–SiC(0001) interaction and, thereby, prevents an identifiable lifting of graphene from the metal surface. The dangling bonds of the defect, however, can form a sufficiently strong bond with the tip apex atom. In addition, a voltage polarity effect was not observed, which renders the involvement of current-induced forces unlikely [64]. Another difference to the previous report concerns the actual behavior of *I*↓ and *I↑*. Within the hysteresis loop, the tunneling current during approach exceeds the current during retraction, *I*↓ *> I*↑, which contrasts the opposite order for graphene on SiC(0001). On the basis of the experimental data alone it is difficult to identify a rationale for this observation. Indeed, electron transport across the junction depends on the tip–graphene and graphene–surface hybridization [23,65]. Therefore, simulations of the non-equilibrium charge transport across the junction are required for a detailed understanding of the observed current traces.

## Conclusion

A combination of STM and AFM experiments unravels the nature of defects in graphene on Ir(111) induced by rare-gas ion bombardment. Defects that are assigned to alleged monatomic vacancy sites by STM measurements represent an intact graphene lattice in AFM topographies. Possibly, a defect in the Ir(111) surface is the origin of the STM-derived contrast. The smallest vacancy defects are represented by triangular structures with four missing C atoms. These tetravacancies reveal an electronic structure clearly different from that of the surrounding intact graphene. Their interior exhibits the tendency to form bonds with the Au probe in close proximity, which is evidenced by shifts of the point of maximum attraction and hysteresis loops in force spectroscopy experiments.

## Supporting Information

File 1 Additional data and figures.

## Data Availability

All data that supports the findings of this study is available in the published article and/or the supporting information to this article. Additional research data is not shared.
