# Supplementary material for: Unveiling the nature of atomic defects in graphene on a metal surface
Source: Beilstein J Nanotechnol. 2024 Apr 15;15:416–25. doi: 10.3762/bjnano.15.37 (PMC11035987; doi:10.3762/bjnano.15.37)
Supplement: File 1 — Additional data and figures. [file Beilstein_J_Nanotechnol-15-416-s001.pdf]

BEILSTEIN JOURNAL OF NANOTECHNOLOGY

Supporting Information

for

Unveiling the nature of atomic defects in graphene on a metal surface

Karl Rothe, Nicolas Néel and Jörg Kröger

Beilstein J. Nanotechnol. **2024,** *15,* 416–425. doi:10.3762/bjnano.15.37

Additional data and figures

License and Terms: This is a supporting information file under the terms of the Creative Commons Attribution License (https://creativecommons.org/licenses/by/4.0). Please note that the reuse, redistribution and reproduction in particular requires that the author(s) and source are credited and that individual graphics may be subject to special legal provisions.
The license is subject to the *Beilstein Journal of Nanotechnology* terms and conditions: (https://www.beilstein-journals.org/bjnano/terms)

Topographies of defect-free graphene

The graphene defects reported in the main article are present only after Ar^+ ion bombardment. Prior to this treatment, clean and defect-free graphene appears in STM topographs (Figure S1).

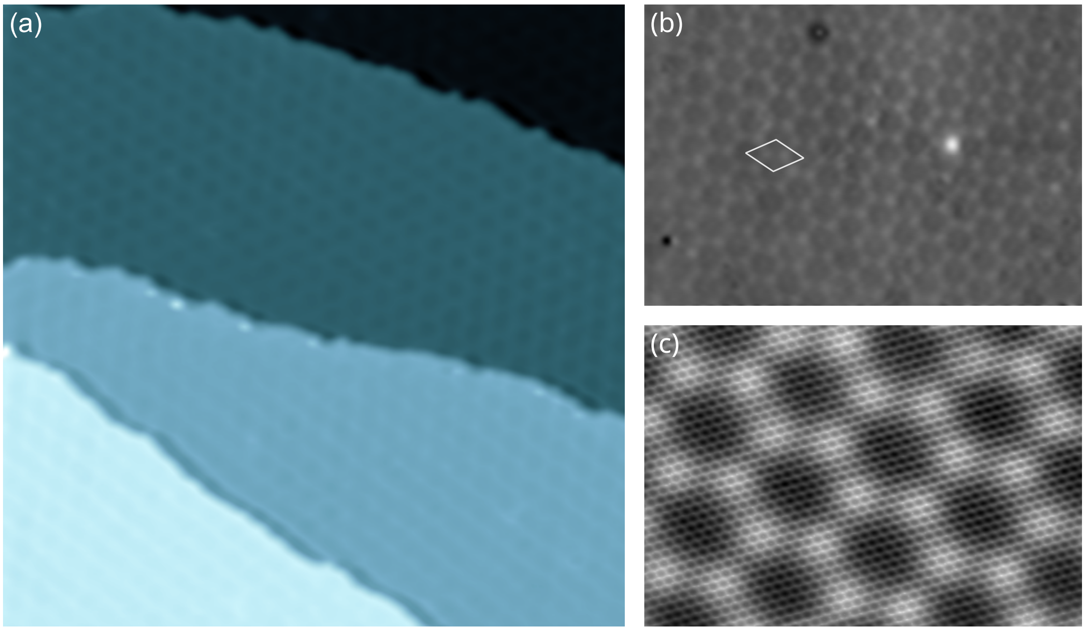

Figure S1: Constant-current STM images of freshly prepared graphene on Ir(111). (a) Four adjacent substrate terraces covered with a single layer of graphene (100 mV, 50 pA, 50 nm × 50 nm). (b) Close-up view of (a) showing the moiré supercell (lozenge) with mounds (dim contrast) and valleys (bright) (1 V, 50 pA, 35 nm × 24 nm). (c) Close-up view of (b) where both the moiré and graphene lattices are visible (10 mV, 5 nA, 5.8 nm × 4 nm).

Tetravacancies at face-centered cubic moiré valleys

The contrast trends in AFM topographs of tetravacancies at hcp moiré valleys are reproduced by tetravacancies at fcc valleys of the moiré lattice. Figure S2a,b shows the contrast inversion in a Δf map of a type-**1** defect at an fcc valley from bright to dim upon increasing the tip excursion towards the surface from **A** to **B** (Figure S2c). Likewise, the evolution of simultaneously recorded constant-height current data for a tetravacancy at an fcc valley (Figure S2d,e) is similar to observations for the vacancy at an hcp valley. In spatially resolved Δf measurements, the point of maximum attraction (F^*) shifts to lower values and to extended tip excursions (Δz^*) upon laterally approaching the defect center (Figure S2f).

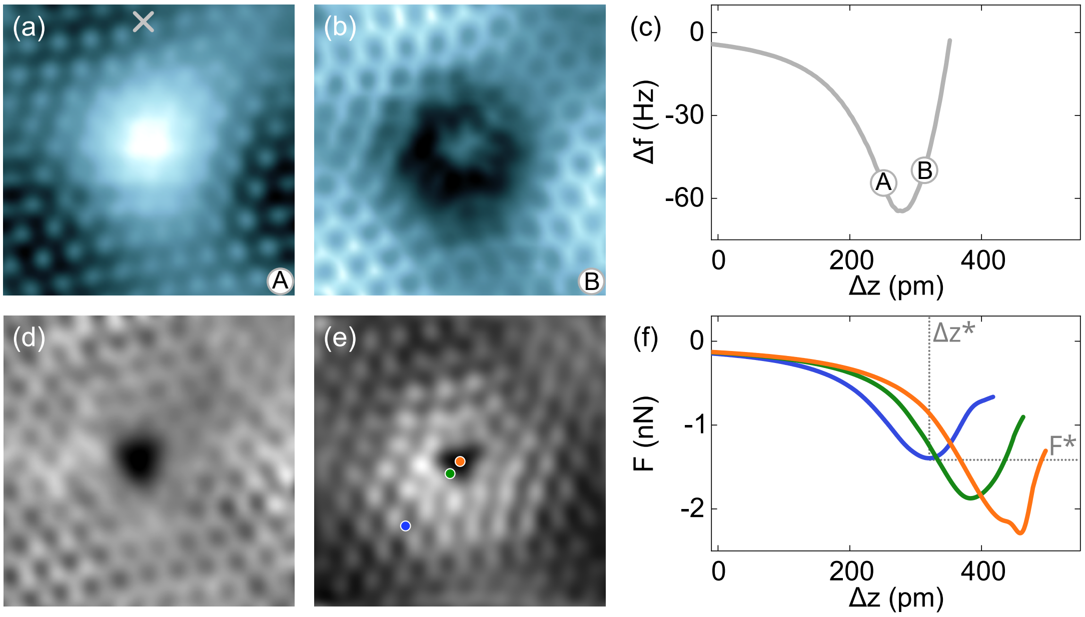

Figure S2: Atomic force and scanning tunneling microscope data for a tetravacancy residing at an face-centered cubic valley of the graphene moiré lattice on Ir(111). (a),(b) Constant-height AFM images (10 mV, 2.4 nm × 2.4 nm) of the defect recorded with tip excursions marked in (c). The gray scale (from dark to bright) covers changes in the resonance frequency change from (a) -83 to -22 Hz and (b) from -106 to 34 Hz. (c) Variation of Δf with tip displacement Δz (tip approach from left to right) on intact graphene (cross in (a)). **A** and **B** mark the tip excursion used for the constant-height Δf maps in (a) and (b), respectively. Zero displacement defines the tip–sample distance at which the feedback loop was deactivated above pristine graphene (10 mV, 50 pA). (d, e) Constant-height tunneling current maps simultaneously recorded with the Δf maps in (a) and (b). The gray scale (from dark to bright) reflects changes in the tunneling current from (d) 5 nA to 23 nA and (e) 41 nA to 130 nA. (f) Total vertical force extracted from $\Delta f(\Delta z)$ data recorded atop the positions marked in (e). Exemplarily, the point of maximum attraction $(\Delta z^*, F^*)$ is indicated for one F trace.

Vertical-force evolution of type-2 defects

In contrast to the behavior of type-**1** defects, defects of the second kind exhibit $F(\Delta z)$ variations that are nearly identical to those obtained from intact graphene. A representative data set is shown in Figure S3, where the STM topograph (Figure S3a) defines the sites for $\Delta f(\Delta z)$ measurements above graphene, the boundary and the center of the defect. The associated total vertical forces are displayed in Figure S3b, where the point of maximum attraction for the three $F(\Delta z)$ traces does not exhibit a similarly pronounced shift in magnitude and position as experienced for the defect of the first kind. This finding is in agreement with the intact graphene lattice associated with type-**2** defects.

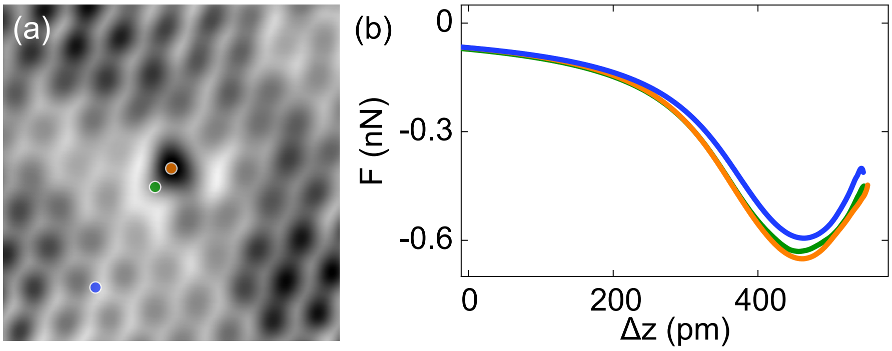

Figure S3: Atomic force spectroscopy data for a type-**2** defect. (a) Constant-current STM image of a type-**2** defect in graphene on Ir(111) (10 mV, 40 nA, 1.8 nm × 1.8 nm). (b) Total vertical force extracted from $\Delta f(\Delta z)$ data recorded atop the positions marked in (a).

Spatial map of hysteresis loop width

To visualize the variation of the hysteresis loop width across the tetravacancy defect, approach and retraction Δf cycles were recorded at various positions atop the defect (crosses in Figure S4a). Loop widths measured within a square area (720 pm × 720 pm) of a regular grid were averaged and transformed into a color (Figure S4b,c). After smoothing with a third-order polynomial, the resulting map shows that the largest loop widths are observed inside the defect close to the defect boundary and center.

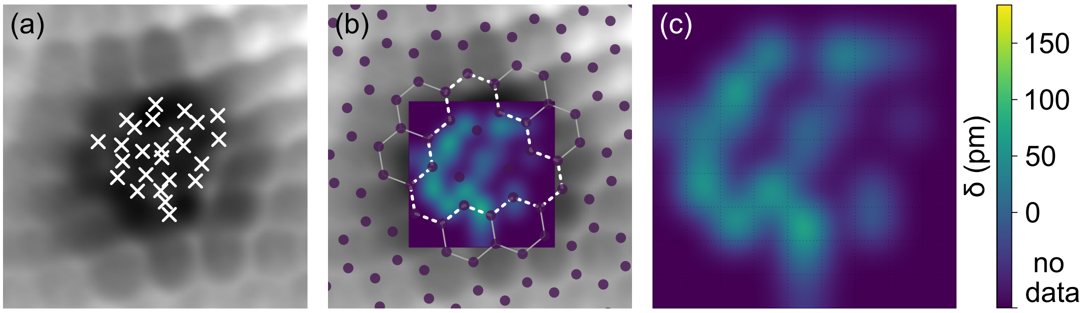

Figure S4: Visualization of spatial hysteresis loop width variation. (a) Constant-height Δf map of a type-**1** defect (10 mV, 1.5 nm × 1.5 nm) with indicated (crosses) positions of $\Delta f\downarrow$ and $\Delta f\uparrow$ measurements. (b) As (a), with superimposed C atom positions (dots) of the graphene lattice and included spatial map of loop widths shown in (c). (c) Map of spatially averaged hysteresis loop widths from Δf approach and retraction traces acquired at the positions marked in (a).
